# Effect of postoperative delirium after cardiovascular surgery on 5-year mortality

**DOI:** 10.1186/s40981-023-00658-0

**Published:** 2023-10-13

**Authors:** Chisaki Yokoyama, Kenji Yoshitnai, Soshiro Ogata, Satsuki Fukushima, Hitoshi Matsuda

**Affiliations:** 1 Department of Anesthesiology, National Cerebral and Cardiovascular Center, 6-1, Kishibeshinmachi, Suita, Osaka Japan; 2 Department of, Preventive Medicine and Epidemiology, National Cerebral and Cardiovascular Center, Suita, Osaka Japan; 3 Department of Cardiovascular Surgery, National Cerebral and Cardiovascular Center, Suita, Osaka Japan

**Keywords:** Postoperative delirium, Cardiovascular surgery, Cardiopulmonary bypass, 5-year mortality

## Abstract

**Introduction:**

Postoperative delirium is a common complication after cardiovascular surgery. A meta-analysis revealed that postoperative delirium was associated with cognitive decline and dementia, which may affect long-term mortality. However, few studies have reported the association between postoperative delirium after cardiovascular surgery and long-term postoperative mortality. Therefore, we investigated the effect of postoperative delirium on 5-year survival rates of patients who underwent cardiovascular surgery.

**Methods:**

We retrospectively reviewed the records of patients who underwent cardiovascular surgery with cardiopulmonary bypass from January 2016 to December 2019. Postoperative delirium was defined as an Intensive Care Delirium Screening score ≥ 3, which might include subclinical delirium. Cox proportional hazards modeling was performed to assess the association between postoperative delirium and mortality. Postoperative mortality in patients with and without delirium was assessed using the Kaplan–Meier method and compared using the log-rank test.

**Results:**

Postoperative delirium was observed in 562 (31.9%) of 1731 patients. There were more elderly patients, more emergent surgery procedures, longer operative time, and larger transfusion volume in the postoperative delirium group. Cox regression analyses showed that delirium (hazard ratio (HR), 1.501; 95% confidence interval (CI), 1.053–2.140; *p* = 0.025) and emergent surgery (HR, 3.380; 95% CI, 2.231–5.122; *p* < 0.001) are significantly associated with 5-year mortality. Among patients who underwent elective surgery, postoperative delirium (HR, 1.987; 95% CI, 1.135–3.481; *p* = 0.016) is significantly associated with 5-year mortality. Kaplan–Meier survival analysis revealed that patients with postoperative delirium had significantly higher 5-year mortality.

**Conclusions:**

Patients with postoperative delirium after cardiovascular surgery have significantly higher 5-year mortality.

## Introduction

Postoperative delirium (POD) is a common complication after cardiovascular surgery; the incidence is 11.4–55% [1–4]. Several studies have reported that POD after non-cardiac surgery is associated with higher postoperative mortality (hazard ratio (HR), 1.95; 95% confidence interval (CI), 1.51–2.52) [5, 6]. However, few studies have evaluated the association between the incidence of POD after cardiovascular surgery and long-term postoperative mortality [7, 8]. These studies had a limited focus on coronary artery bypass grafting (CABG) or 1-year mortality. Furthermore, a recent meta-analysis demonstrated that POD is also associated with a significant risk for dementia (odds ratio (OR), 6.08; 95% CI, 3.80–9.72; *I*^2^ = 0) and the risk trajectory for cognitive decline associated with POD within 5 years after surgery grows exponentially [9]. The impact of POD on long-term mortality has increasingly important implications in an aging society. Therefore, we examined the relationship between POD and 5-year mortality because prolonged POD might lead to dementia, which might be related to mortality [10, 11].

## Methods

We retrospectively reviewed the clinical records of 2003 patients who underwent cardiovascular surgery with CPB between January 2016 and December 2019. Patient data were extracted from electronic medical records.

The inclusion criteria were the age of 15 years or older and Japanese as the native language. Patients were excluded if they met any of the following criteria: preoperative delirium; preoperative history of dementia, corticobasal degeneration, or psychological disorder (depression, schizophrenia, or alcoholism); and lack of Intensive Care Delirium Screening Checklist (ICDSC) [12] data. The ethics review board of the National Cerebral and Cardiovascular Center approved the study protocol on January 21, 2021. The need for written informed consent was waived due to the study’s retrospective nature.

We collected data on the following clinical characteristics: age, sex, height, body weight, body mass index (BMI), diagnosis, surgical procedure, operative time, CPB time, intraoperative blood loss, intraoperative transfusion volume, duration of intensive care unit (ICU) stays, total amount of fentanyl, dexmedetomidine in ICU, and propofol in ICU; duration of hospital stays; ICDSC score; comorbidities; and 5-year postoperative outcome. POD was defined as ICDSC score ≥ 3, which might include subclinical delirium [13, 14]. The ICDSC is a tool used to assess and screen for delirium in patients. The ICDSC comprises eight items that evaluate different symptoms of delirium. Each item can score 0 (absent) or 1 (present). Here are the eight items: altered level of consciousness; inattention; disorientation; hallucination, delusion, or psychosis; psychomotor agitation or retardation; inappropriate speech or mood; sleep/wake cycle disturbances; and symptom fluctuation. ICDSC scores were recorded during the ICU stay by nurses involved in the patient’s daily clinical care in the ICU twice daily. ICDSC score ≥ 4 is still used to screen for delirium, but Soenke and colleagues demonstrated that the cutoff of ≥ 3 has a higher agreement with the Diagnostic and Statistical of Mental Disorder, Fourth Edition, Text Revision (DSM-IV-TR) (Cohen’s κ, 0.68, indicating substantial agreement) than the cutoff of ≥ 4 (Cohen’s κ, 0.59, indicating medium agreement) [13]. Therefore, we also tested whether the patients in the highest maximum ICDSC score tertile had higher 5-year mortality than patients in the other tertile.

### Statistical analysis

We assessed the association between POD and 5-year mortality and evaluated risk factors for POD. Categorical variables were evaluated using the chi-square test. Continuous variables were assessed using the Mann–Whitney *U* test. Cox proportional hazards modeling was used to evaluate the association between POD and mortality after adjusting the following possible confounders included in Euroscore II for calculating mortality: age, sex, serum creatinine, severe neurological dysfunction, recent myocardial infarction, left ventricular ejection fraction, redo surgery, emergent surgery, and cardiac surgery procedure type [15]. Emergent surgery is potentially a strong risk factor for mortality. Therefore, we also performed a stratified analysis based on elective surgery to assess whether delirium affects mortality even after elective surgery, when the patient’s clinical condition was stable. To clarify the impact of the incidence of delirium on long-term outcomes for each cardiovascular surgery procedure type, we performed a subgroup analysis for each surgery procedure using Cox regression analysis adjusting the same possible confounders. Postoperative mortality was assessed using the Kaplan–Meier method and compared using the log-rank test. Risk factors for POD were also evaluated using logistic regression to understand the background of POD. All statistical analyses were performed using EZR version 1.53, a modified version of the R commander designed to add statistical functions frequently used in biostatistics, or Stata Standard Edition, version 17 (StataCorp, College Station, TX, USA); *p* < 0.05 was considered statistically significant.

### Sample size calculation

Based on the mortality associated with cardiovascular surgery in patients with delirium (21.8%) and without delirium (8.7%) in a previous study [16], we use an observational period of 5 years and a study entry period of 3 years, based on alpha of 0.05 and 1-beta of 0.8. The sample size, calculated with EZR, was 101 patients in each group. If the mortality rate was 21.8% and 100 events occurred, 467 people are needed to include 10 covariates in the analysis. We collected 1700 patients to test our hypothesis more accurately after considering the possibility of unexpected dropouts due to the retrospective nature of the study. Risk factors for POD were assessed using multivariate logistic regression.

## Results

This study included 1762 patients. Due to the lack of ICD-SC score, 241 patients were excluded. The median observation time was 1030.5 (interquartile range (IQR), 735–1354) days. POD was observed in 526 (29.9%) patients. Death occurred in 150 (8.5%) patients.

The clinical characteristics of the study patients are summarized in Table 1. Compared to patients without delirium, there were more elderly patients in the delirium group. In addition, the proportion of patients who underwent emergent surgery, operative time, and transfusion volume were higher in the delirium group. The proportion of patients with POD was significantly lower among those who underwent adult congenital heart surgery, valve surgery, aortic surgery, or minimum invasive cardiac surgery (MICS) surgery, and significantly higher among those who underwent pulmonary endarterectomy for chronic thromboembolic pulmonary hypertension. Patients with POD received a significantly higher total amount of dexmedetomidine and propofol in ICU, respectively, than those without POD.

**Table 1:** Characteristics of patients with and without postoperative delirium

	No delirium (*n* = 1236)	Delirium (*n* = 526)	*p* value
Age (years)	64 [48, 73]	771 [60, 78]	< 0.001
Male sex	727 (58.8%)	292 (55.5%)	0.200
Height (cm)	163.0 [155, 170]	161.0 [153.5, 168.0]	0.007
Weight (kg)	58.0 [50.0, 67.6]	59.7 [50.0, 67.6]	0.998
Operative time (min)	275 [207, 379]	341 [250, 445]	< 0.001
Emergent surgery	326 (26.4%)	229 (43.5%)	< 0.001
Transfusion (mL)	1,320 [750, 2050]	2,010 [1315, 2580]	< 0.001
Cerebral infarction	229 (18.5%)	115 (21.9%)	0.11
Hypertension	527 (42.6%)	261 (46.9%)	0.007
Pulmonary hypertension	119 (9.6%)	54 (10.3%)	0.680
Diabetes mellitus	532 (43.0%)	177 (33.7%)	< 0.001
Atrial fibrillation	341 (27.6%)	150 (28.5%)	0.690
Adult congenital heart disease	40 (3.2%)	2 (0.4%)	< 0.001
Valvular disease	445 (36.0%)	142 (27.0%)	< 0.001
Aortic disease	270 (21.8%)	186 (35.4%)	< 0.001
Isolated CABG	52 (4.2%)	29 (5.5%)	0.230
VAD or heart transplantation	163 (13.2%)	64 (12.2%)	0.560
MICS	134 (10.8%)	20 (3.8%)	< 0.001
Redo surgery	78 (6.3%)	42 (8.0%)	0.200
Pulmonary thromboendarterectomy	14 (1.1%)	23 (4.4%)	< 0.001
Intracardiac mass	28 (2.3%)	10 (1.9%)	0.630
Other	12 (1.0%)	8 (1.5%)	0.320
Fentanyl (mL, *n* = 169)	94.7 (48.3, 306)	88.3 (42.5, 220.6)	0.150
Dexmedetomidine in ICU (mL, *n* = 1263)	21.8 (11.1, 52.8)	28.9 (12.3, 72.1)	0.014
Propofol in ICU (mL, *n* = 1731)	67.6 (30.3, 172.5)	151.4 (56, 312.9)	< 0.001
ICU stay (days)	3 (2, 5)	5 (3, 9)	< 0.001

Data are expressed as medians [interquartile range]

*CABG* coronary artery bypass grafting, *ICU* intensive care unit, *MICS* minimally invasive cardiac surgery, *VAD* ventricular assist device

Cox regression results for mortality are shown in Table 2. POD (HR, 1.501; 95% CI, 1.053–2.140; *p* = 0.025), age (HR, 1.018; 95% CI, 1.005–1.032; *p* = 0.009), operative time (HR, 1.002; 95% CI, 1.000–1.003; *p* = 0.023), hypertension (HR, 2.048; 95% CI, 1.300–3.225; *p* = 0.002), emergent surgery (HR, 3.380; 95% CI, 2.231–5.122; *p* < 0.001), creatinine (HR, 1.190; 95% CI, 1.093–1.295; *p* < 0.001), and total transfusion volume (HR, 1.036; 95% CI, 1.022–1.051; *p* < 0.001) were significantly associated with higher 5-year mortality. Valvular disease (HR, 0.443; 95% CI, 0.248–0.790; *p* = 0.006) and aortic disease (HR, 0.339; 95% CI, 0.185–0.621; < 0.001) were associated with lower 5-year mortality.

**Table 2:** Cox proportional hazards regression results for 5-year mortality

	Hazard ratio	Standard error	*p* value	95% CI lower limit	95% CI upper limit
Delirium	1.501	0.272	0.025	1.053	2.140
Age	1.018	0.007	0.009	1.005	1.032
Sex	0.766	0.140	0.146	0.535	1.097
Operative time	1.002	0.001	0.023	1.000	1.003
Atrial fibrillation	0.859	0.174	0.453	0.578	1.278
Diabetes mellitus	1.574	0.366	0.051	0.998	2.482
Hypertension	2.048	0.475	0.002	1.300	3.225
Emergency surgery	3.380	0.717	< 0.001	2.231	5.122
Creatinine	1.190	0.052	< 0.001	1.093	1.295
Cerebral infarction	1.258	0.254	0.255	0.847	1.869
Valvular disease	0.443	0.131	0.006	0.248	0.790
Aortic disease	0.339	0.105	< 0.001	0.185	0.621
Adult congenital heart disease	1.032	0.606	0.958	0.326	3.264
Redo surgery	0.554	0.196	0.095	0.278	1.108
Pulmonary thromboendarterectomy	0.734	0.560	0.685	0.165	3.271
Isolated CABG	0.789	0.313	0.549	0.363	1.715
Total transfusion volume	1.035	0.008	< 0.001	1.020	1.050

*CABG* coronary artery bypass grafting, *CI* confidence interval

**Fig. 1:**
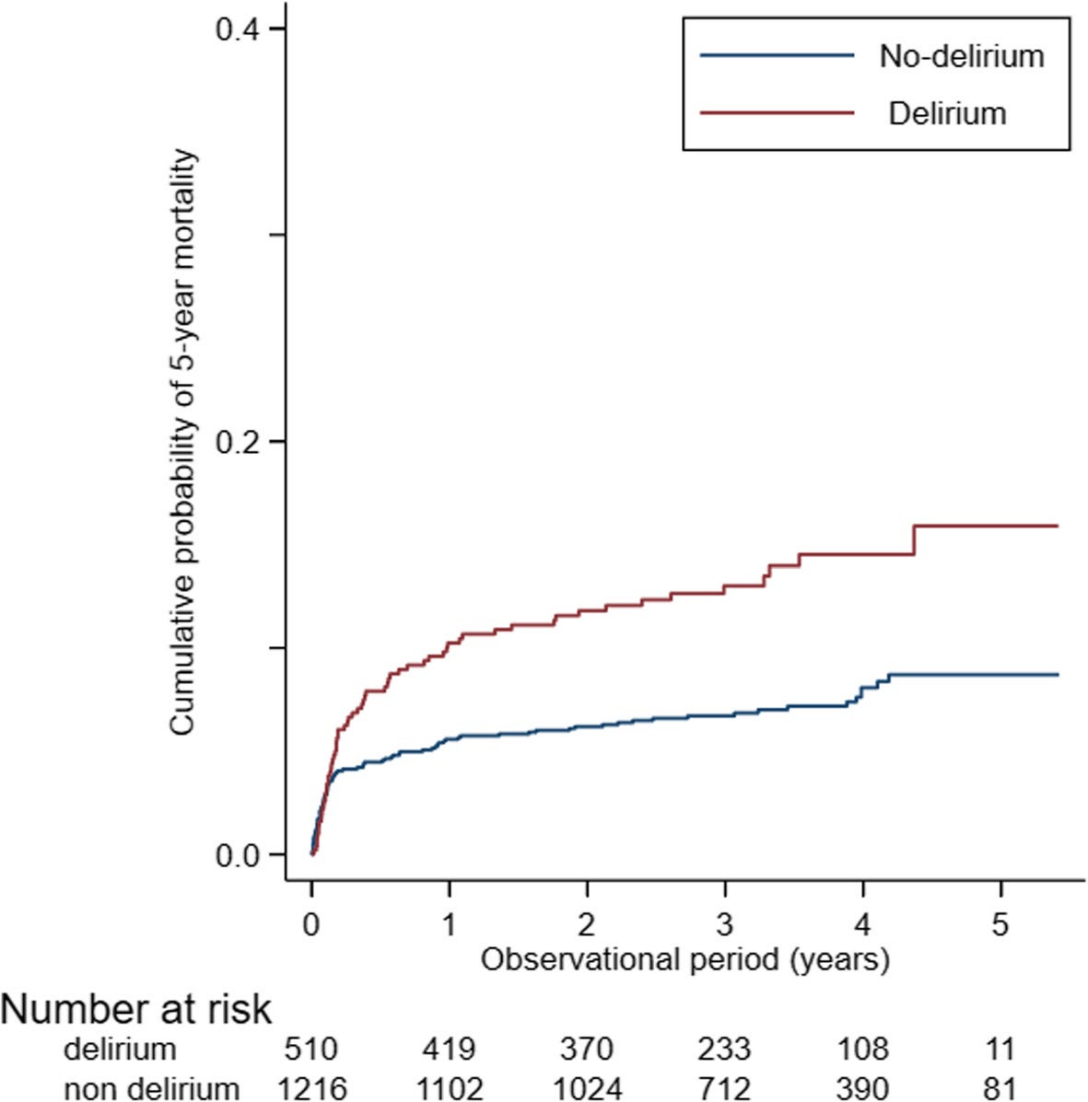
Five-year mortality of patients with and without postoperative delirium after cardiovascular surgery

Cox regression results for mortality in analyses restricted to patients who underwent elective surgery are shown in Table 3. Delirium (HR, 1.987; 95% CI, 1.135–3.481; *p* = 0.016), operative time (HR, 1.003; 95% CI, 1.001–1.005; *p* = 0.007), hypertension (HR, 2.048; 95% CI, 1.300–3.225; *p* = 0.002), creatinine (HR, 1.217; 95% CI, 1.097–1.351; *p* < 0.001), and total transfusion volume (HR, 1.024; 95% CI, 1.001–1.048; *p* = 0.043) were significantly associated with higher 5-year mortality. Kaplan–Meier survival analysis revealed higher 5-year mortality in the delirium group (Fig. 1).

**Table 3:** Cox proportional hazards regression results for 5-year mortality among patients who underwent elective surgery

	Hazard ratio	Standard error	*p* value	95% CI lower limit	95% CI upper limit
Delirium	1.987	0.568	0.016	1.135	3.481
Age	1.006	0.011	0.560	0.986	1.027
Sex	0.772	0.219	0.362	0.442	1.347
Operative time	1.003	0.001	0.007	1.001	1.005
Atrial fibrillation	1.21	0.377	0.541	0.657	2.229
Diabetes mellitus	1.038	0.372	0.918	0.514	2.095
Hypertension	1.796	0.611	0.085	0.922	3.499
Creatinine	1.217	0.065	< 0.001	1.097	1.351
Cerebral infarction	0.842	0.32	0.652	0.4	1.774
Valvular disease	0.551	0.243	0.177	0.232	1.309
Aortic disease	0.364	0.169	0.030	0.146	0.905
Adult congenital heart disease	0.332	0.291	0.208	0.060	1.849
Redo surgery	0.93	0.47	0.887	0.345	2.507
Pulmonary thromboendarterectomy	0.333	0.364	0.314	0.039	2.833
Isolated CABG	0.981	0.625	0.976	0.282	3.418
Total transfusion volume	1.024	0.012	0.043	1.001	1.048

*CABG* Coronary artery bypass grafting, *CI* Confidence interval

Table 4 shows the risk factors for POD. Multivariate logistic regression revealed that age (odds ratio (OR), 1.031; 95% CI, 1.021–1.042; *p* < 0.001), emergent surgery (OR, 1.587; 95% CI, 1.111–2.266; *p* = 0.008), and operative time (OR, 1.002; 95% CI, 1.001–1.003; *p* = 0.001) are significantly associated with a higher incidence of POD. Surgery for adult congenital heart disease (OR, 0.137; 95% CI, 0.023–0.819; *p* = 0.029) was associated with a lower incidence of POD.

**Table 4:** Risk factors of postoperative delirium

	Odds ratio	*p* value	95% CI lower limit	95% CI upper limit
Age	1.031	< 0.001	1.021	1.042
Sex	0.864	0.263	0.668	1.116
Operative time	1.002	0.001	1.001	1.003
Emergent surgery	1.587	0.011	1.111	2.266
Total transfusion volume	1.000	0.263	1.000	1.000
Cerebral infarction	1.184	0.272	0.876	1.6
Hypertension	1.097	0.556	0.807	1.49
Diabetes mellitus	0.907	0.522	0.672	1.223
Atrial fibrillation	1.153	0.305	0.878	1.515
Creatinine	1.224	< 0.001	1.115	1.343
Adult congenital heart disease	0.137	0.029	0.023	0.819
Valve disease	0.612	0.352	0.218	1.720
Aortic disease	0.742	0.576	0.26	2.112
Isolated CABG	0.732	0.589	0.235	2.272
LVAD and heart transplantation	0.876	0.804	0.308	2.489
Minimally invasive cardiac surgery	0.503	0.232	0.163	1.552
Redo surgery	0.939	0.909	0.32	2.758
Pulmonary endarterectomy	2.966	0.097	0.821	10.719
Intracardiac mass	0.594	0.432	0.162	2.178
Fentanyl	0.999	0.106	0.998	1.000
Dexmedetomidine in ICU	1.000	0.747	0.999	1.001
Propofol in ICU	1.000	0.118	1.000	1.000

*CABG* Coronary artery bypass grafting, *CI* Confidence interval, *ICU* Intensive care unit, *LVAD* Left ventricular assist device

## Discussion

We found that patients with POD after cardiovascular surgery have significantly higher 5-year mortality. Emergent surgery, operative time, creatinine, and transfusion volume were also significantly associated with higher 5-year mortality. Hypertension, valvular disease, and aortic disease were associated with lower 5-year mortality. In patients who underwent elective surgery, POD was also associated with 5-year mortality. Age, emergent surgery, and operative time were associated with a higher incidence of POD. Adult congenital heart disease was associated with a lower incidence of POD.

Dubiel and colleagues reported that POD in patients who undergo cardiac surgery is significantly associated with 1-year functional survival, defined as requiring admission to a long-term care facility or death. The HR for delirium was 2.58 (95% CI, 1.20–5.54) [7]. Billie-Jean and colleagues also reported that in patients who underwent CABG, POD is associated with long-term mortality; the HR for delirium was 1.52 (95% CI, 1.29–1.78) [8]. We demonstrated that patients with POD have a significantly higher risk of mortality after various types of cardiovascular surgery, including CABG, surgery for valvular disease, minimally invasive cardiac surgery, and left ventricular assist device placement. The adjusted HR of POD was 1.501 (95% CI, 1.053–2.140). A previous systematic review and meta-analysis reported that POD after non-cardiac surgery is associated with a four-fold increase in the odds of death (odds ratio, 4.12; 95% CI, 3.29–5.15) [16]. We demonstrated that POD is also associated with 5-year mortality after various types of cardiovascular surgery. Previously, postoperative mortality for cardiac surgery was reported as 1.3% (IQR, 0.2–2.2%) for CABG and 3.1% (IQR, 1.7–5.1%) for valve procedures, which are higher than postoperative mortality for non-cardiac surgery [17]. Therefore, the additional effects of POD on postoperative mortality might be smaller than for non-cardiac surgery.

ICDSC has been used as a screening test for delirium. Whether to use a cutoff value of 3 or 4 remains controversial [13, 14]. We used a cutoff value of 3 as subclinical delirium that may be useful to prevent the underestimation of postoperative delirium.

Surgical procedure type did not affect 5-year mortality. Minimally invasive cardiac surgery facilitated earlier return to work [18] but did not reduce 5-year mortality. The reason is unknown, but other factors might overcome the benefit of the minimally invasive nature of the procedure. Aortic surgery was paradoxically associated with lower 5-year mortality in elective cardiac surgery. The cause was also unknown, but patients with aortic surgery have relatively preserved cardiac function, which might contribute to the low 5-year mortality rate.

Our aim was to assess whether POD has an impact on 5-year mortality after cardiac surgery. Risk factors for POD were also investigated to assess the effect of POD on 5-year mortality. Risk factors for POD included age, emergent surgery, operative time, and creatinine. In previous systematic reviews, age and preoperative cognitive or psychiatric conditions were reported as risk factors for POD [19–21]; our findings are compatible. Previous studies reported that POD after cardiac surgery is associated with white matter lesions, which were observed in patients with aged and damaged brains [22–24]. Whether POD is a surrogate for having an aged and damaged brain and whether perioperative therapy for POD can improve the prognosis of patients after cardiovascular surgery were questions that arose from this study. Emergent surgery and operative time were associated with POD. Emergent surgery is associated with a high risk for POD, probably due to the lack of adjustment for certain environmental conditions such as lighting and temperature. Ensuring patient comfort has proven to be highly effective in preventing POD in the elective surgery setting [25]. Operative time is an intraoperative factor that might be associated with the incidence of POD. Longer operative time means longer CPB time, which leads to systemic inflammation. CPB induces an increase in blood–brain barrier (BBB) permeability, resulting in cognitive dysfunction [26]. After CPB, glial activation occurred in a rat model [27], which might have been induced by increased BBB permeability. BBB permeability is likely to be higher in damaged brains with white matter lesions, which might also be prone to neuroinflammation. Chronic neuroinflammation could lead to prolonged cognitive decline and dementia, resulting in a worse prognosis.

This study has several limitations. First, this study was retrospective. The Kaplan–Meier survival analysis included the impact of age, emergent surgery, and operative time. However, after adjusting the confounders, postoperative delirium still remained significantly associated with 5-year mortality. We could not collect the cause of death due to the lack of form of the cause of death in electronic medical records. Therefore, we could not discuss the association between POD and mortality directly. However, we may need to consider the effect of postoperative delirium on long-term mortality. Although we adjusted for possible confounders using Cox regression analysis, the results might have been influenced by unadjusted confounders. Second, we can only assess a patient’s delirium status with the ICDSC when the patient is awake in the ICU. In other words, it was impossible to evaluate comatose patients with the ICDSC. This characteristic of the ICDSC leads to the exclusion of patients with severe disorders of consciousness, which consequently biases our data. Also, ICDSC was not performed after moving to the general ward, which may lead to underestimating the incidence of postoperative delirium. However, most patients were transferred to the general ward after confirming that patients were free from delirium. Therefore, the risk of underestimation may be low. Third, data on preoperative medications prior to emergency surgery was not available. The large amount of missing data might have affected the validity of the findings. However, missing data might have had little impact on the results because patients receiving medications that could be associated with POD, such as dementia or psychological disorders, were excluded. Fourth, we did not access mild cognitive impairment preoperatively, which may impact postoperative delirium and mortality. However, the assessment of mild cognitive impairment has some difficulty due to the lack of a gold standard of diagnosis. This is a point to consider in any new prospective study.

## Conclusion

Patients with POD after cardiovascular surgery involving CPB have significantly higher 5-year mortality. Our results showed that the incidence of postoperative delirium was associated with the 5-year mortality after cardiac surgery.

## Data Availability

The datasets used and/or analyzed during the current study are available from the corresponding author on reasonable request.

## Abbreviations

BMI: Body mass index
CABG: Coronary artery bypass grafting
CI: Confidence interval
CPB: Cardiopulmonary bypass
HR: Hazard ratio
ICU: Intensive care unit
ICDSC: Intensive Care Delirium Screening Checklist
IQR: Interquartile range
MICS: Minimally invasive cardiac surgery
OR: Odds ratio
POD: Postoperative delirium
